# Polo-Like Kinase 2: From Principle to Practice

**DOI:** 10.3389/fonc.2022.956225

**Published:** 2022-07-08

**Authors:** Chuanyong Zhang, Chuangye Ni, Hao Lu

**Affiliations:** ^1^ Hepatobiliary Center, The First Affiliated Hospital of Nanjing Medical University, Nanjing, China; ^2^ Key Laboratory of Liver Transplantation, Chinese Academy of Medical Sciences, Nanjing, China

**Keywords:** polo-like kinase 2, cell cycle, stress, tumor, neurodegenerative disease

## Abstract

Polo-like kinase (PLK) 2 is an evolutionarily conserved serine/threonine kinase that shares the n-terminal kinase catalytic domain and the C-terminal Polo Box Domain (PBD) with other members of the PLKs family. In the last two decades, mounting studies have focused on this and tried to clarify its role in many aspects. PLK2 is essential for mitotic centriole replication and meiotic chromatin pairing, synapsis, and crossing-over in the cell cycle; Loss of PLK2 function results in cell cycle disorders and developmental retardation. PLK2 is also involved in regulating cell differentiation and maintaining neural homeostasis. In the process of various stimuli-induced stress, including oxidative and endoplasmic reticulum, PLK2 may promote survival or apoptosis depending on the intensity of stimulation and the degree of cell damage. However, the role of PLK2 in immunity to viral infection has been studied far less than that of other family members. Because PLK2 is extensively and deeply involved in normal physiological functions and pathophysiological mechanisms of cells, its role in diseases is increasingly being paid attention to. The effect of PLK2 in inhibiting hematological tumors and fibrotic diseases, as well as participating in neurodegenerative diseases, has been gradually recognized. However, the research results in solid organ tumors show contradictory results. In addition, preliminary studies using PLK2 as a disease predictor and therapeutic target have yielded some exciting and promising results. More research will help people better understand PLK2 from principle to practice.

## Introduction

Polo-like kinase (PLK) 2 is one of PLKs, a family of serine/threonine kinases. PLK2 shares the conserved N-terminal kinase catalytic domain and one or two C-terminal Polo box domains (PBD) with its siblings (PLK1,3-5) (1, 2). The PBD of PLK2 consists of 218 amino acid residues, including two 12-chain β sandwich conserved domains formed by β6α structures consisting of 30 amino acid residues (3–5). PLK2 plays an important role in many aspects, e.g., cell cycle (6–8), cell differentiation (9–11), ontogenesis (12), stress response (13), tumorigenesis (14), neurodegenerative diseases (15–17), inflammation and injury (18).

The function of PLK2 is regulated by many mechanisms. Histone deacetylase inhibitor trichostatin A (TSA) could induce upregulated PLK2 expression in human osteosarcoma cell line (MG-63), which may be resulted from TSA-induced GATA-1 acetylation enhancing its DNA-binding ability and initiating the PLK2 promoter, indicating acetylation promoting PLK2 expression (19). Acetylation of PLK2 prohibits the degradation by ubiquitination and participates in centriole replication at the appropriate time (20). Promoter methylation induced by hypoxia and tumor downregulates the PLK2 expression, involved in development and progression of diseases (21–26). Both E3 ubiquitin ligase RNF180 (ring finger protein 180) (27) and miR-101-3p target gene SKP1 (S-phase kinase-associated protein 1) (28) might interact with PLK2 and induce its ubiquitination and degradation. Downregulation of miR-27b in oral lichen planus reduces its inhibition to PLK2 3’untranslated region, leading to proliferation of human oral keratinocytes (29). Nuclear factor erythroid 2-related factor 2 (Nrf2) activated lncRNA (Nrf2-lncRNA) is a competing endogenous RNA of PLK2 and cyclin-dependent kinase inhibitor 1 (p21^cip1^), which induces PLK2/Nrf2/p21^cip1^ to complexate and activate Nrf2 during p53 activation by binding to miR-128 and miR-224, facilitating translation of PLK2 and p21^cip1^ (30). Starvation results in the elevated androgen production and depresses PLK2 expression, while relationship between PLK2 and steroid metabolism remains unclear (31). Transcription factor Sp1 plays an important role in the upregulation of PLK2 stimulated by hCG in cultured rat granulosa cells (32). Chemical carcinogens (33) and γ radiation (34) could also increase the PLK2 expression.

However, there are still many deficiencies in the current understanding of PLK2. For example, studies on PLK2 in microbial infection and immunity, and fibrotic diseases are still insufficient. Its role in hematological neoplasma, solid organ tumor and neurodegenerative diseases is also controversial. Here, we summarize the roles of PLK2 in mammalian cell cycle and non-cell cycle signaling pathways, hoping to provide help for further study of PLK2.

## Role of PLK2 in Normal Physiological Processes

### Mitosis

Mitosis is the process by which eukaryotic cells divide to produce their progeny. The entire process from the completion of one division to the end of the next is called the cell cycle, which consists of interphase and mitotic (M) phase. The interphase could be divided into G1 phase, S phase and G2 phase, in which DNA replication and protein synthesis finishes. During the M phase, the genetic material in the nucleus and organelles are split in a specific way to form progeny cells. Some of these cells continue to enter G1 phase and start the next round of mitosis. Others enter the G0 phase, where the cell cycle stagnates, but can re-enter the G1 phase to replicate after appropriate stimulation. PLK2 expresses in G1 phase; silencing of PLK2 results in the growth retardation and delays S phase transition in embryonic fibroblasts and placental dysplasia in mice, revealing that PLK2 if not essential, but plays a critical role at least in mammalian growth and development (35). Significantly up-regulated PLK2 expression stimulates centriole replication in human, pig, and sheep parthenogenetic cell lines (36). As a target, PLK2 could be induced by wild-type p53; inhibition with siRNA causes mitotic catastrophe in paclitaxel-exposed cells (37). High expressed PLK2 in breast tissue regulates the orientation of mitotic spindle and maintains the polarity of ductal epithelial cells (6). When breast cancer cell line MCF-7 is exposed to zinc, expression of PLK2 is dramatically reduced, leading to cell cycle arrest and cancer cell adaption (38). In rats, PLK2 is highly induced in ovarian granulosa cells; overexpressed PLK2 blocks the cell cycle in the G0/G1 phase, while downregulation of it decreases the number of G0/G1 phase cells but increases the cell vitality (32). So, effects of PLK2 on G0/G1 phase transition depends on cell type.

In mammalian cells, centrosome replication is a hallmark of mitosis, starting from G1/S transition and finishing till S/G2 (39). Activation of PLK2 in G1/S transition is essential to centriole replication and centrosome correlation, which is important for cell replication (7, 8). Mutation of PBD prohibits centriole localization and hampers centriole replication (8). PLK2 is acetylated in the process of promoting centrosome replication, which protects PLK2 from ubiquitination degradation. The deacetylase Sirtuin 1 (SIRT1) acting as a temporal regulator, is phosphorylated and activated in early and middle G1 phase promoting deacetylation and degradation and dephosphorylated itself in late G1 phase leading to a reduced PLK2 affinity and rapid PLK2 accumulation, which contributes to the timely initiation of centriole replication (20). PLK2 catalyzes the phosphorylation of S589 and S595 residues in centrosomal P4.1-associated protein (CPAP), which is crucial for the formation of procentriole; CPAP is phosphorylated in a cell cycle stage-specific manner, increasing during the G1/S transition and decreasing at the end of mitosis. Phosphorylated CPAP is preferentially located in the procentriole. Overexpression of an anti-phosphorylated CPAP mutant fails to form elongated centrioles (40).

Cell cycle regulation by PLK2 is co-regulated by CDK2/Cyclin E, CDK2/Cyclin A complex and PLK4 (7, 41). Expression of PLK2 in rat ovary is induced by hCG; prostaglandin and EGF signaling pathways are involved in regulating PLK2 expression; and the transcription factor Sp1 plays an important role in the upregulation of PLK2 (32). PLK2 regulates centrosome replication through polo-box-dependent binding of NPM (nucleophosmin)/B23 and phosphorylation of Ser4 at the S phase (42). Mis-regulation resulted from PLK2 dysfunction is the most likely cause of changes in chromosome segregation, presence of multiple polymeric functional centrosomes, and mass cell death in embryonic stem cells with beta-catenin deletion (43). Centrosome amplification is considered a main cause of chromosome instability in cancer cells. One of the mechanisms is overreplication of centrosomes within a single cell cycle. Rho-associated kinases (ROCK2), PLK2 and PLK4 are essential for centrosome duplication in cells blocked by DNA synthesis inhibitors; In the centrosome amplification rescue assay, PLK2 indirectly activates ROCK2 by phosphorylation of NPM, while PLK4 acts downstream of ROCK2 to drive and block centrosome amplification in cells (44).

### Meiosis

Meiosis is needed for sexual reproduction. Within this process, the DNA replicates once but the cell divides twice, resulting in four progenies with half the number of chromosomes. In C. elegans, pairing and synapsis of homologous chromosome rely on pairing centers (PCs), which locates in special regions at the end of chromosomes and interacts with the nuclear membrane and cytoplasmic microtubules; at the onset of meiosis, PCs recruits PLK2 in response to ZIM/Him-8, a zinc finger protein, to induce nuclear membrane remodeling, chromosome pairing and synapsis (45–47). PLK2 is involved in the establishment of meiotic specific SUN-1 phosphorylation and SUN/KASH dynamic regulation (47). During meiosis, the conserved SUN/KASH nuclear membrane bridge establishes a transient link between chromosome ends and the cytoskeleton, which ensures homologous chromosome aggregation and avoids non-homologous pairing. During pairing and recombination, chromosomal movement begins and SUN-1 aggregates at the chromosomal ends associated with the nuclear membrane and is phosphorylated in a CHK2 - and PLK2-dependent manner. While meiosis is incomplete, PLK-2 continues to be recruited to the chromosome ends in a sun-1-phosphorylation-dependent manner that is required to characterize continuous chromosome movement and zygotic line stop. Chromosomal pairing (synapsis) requires SUN-1 phosphorylation (48). In addition, PLK2 and phosphorylated SYP-1 ensure the generation of short-arm subdomains and facilitates chromosome segregation in meiosis I (49). PLK2 also mediates cell cycle delay and the apoptosis with unsuccessful synapsis of nuclear chromosomes. Functional defects caused by PLK2 knockout (KO) or mutation can lead to meiosis chromosome pairing and synapsis failure (45, 47). PLK2 plays an indispensable role in the successful completion of meiosis.

### Cell Differentiation

PLK2 also plays a vital role in cell differentiation in addition to cell cycle. According to zebrafish model and human umbilical vein endothelial cell (HUVEC) culture, loss of PLK2 function results in a reduction in cell sprouting and migration, while overexpression promotes angiogenesis; PLK2 controls angiogenesis by binding PDZ-GEF and regulating RAP1 activity during endothelial cell lamellipodia formation and extracellular matrix attachment; Constitutively activated RAP1 could reverse endothelial growth defects in PLK2 KO zebrafish and HUVEC (9). Lineage negative bone marrow cells (lin-BMCs) are enriched in endothelial progenitor cells and mediate vascular repair, whose number and function decrease in an age-dependent manner. PLK2 in lin-BMCs is negatively regulated by miR-146a, that is, overexpression of miR-146a in young lin-BMCs inhibits PLK2 expression, resulting in increased aging, apoptosis and impaired angiogenesis through p16Ink4a/p19Arf and p53, respectively. Inhibition of miR-146a in aged lin-BMCs increases PLK2 expression and rejuvenates lin-BMCs, leading to reduced senescence and apoptosis, thereby promoting angiogenesis (10). As a new identified target of miR-126-3p, PLK2 also plays a regulating role in perivascular cells (PVC) and perivascular matrix.

miR-126-3p inhibits the expression of target genes PLK2 and SPRED1 and induces the phosphorylation of extracellular signal-regulated kinase (ERK) 1/2 to stimulate the expression of TLR3, thus regulating the cell-cell and cell matrix contact of PVC, promoting the conversion of immature blood vessels into mature and less permeable blood vessels. Inhibition of PLK2 and SPRED1 expression could mimic the effect of miR-126-3p in PVC but has no effect on the phosphorylation of ERK1/2, suggesting that PLK2 inhibits perivascular matrix formation in an ERK-independent manner (11). Laminin (LN) slows the proliferation of cardiac progenitor cells (CPC), induces the expression of cardiac lineage-specific genes, and promotes the endothelioid differentiation of CPC. After CPC is cultured on LN, YAP (Yes-associated protein) phosphorylation (Ser127) increases, which is confined to the cytoplasm and rapidly degraded by proteasome, thereby inhibiting cell proliferation. As a possible downstream effector, the mRNA level of PLK2 depends on the stability of YAP. Downregulation of PLK2 expression might simulate CPC performance observed in LN, while overexpression of PLK2 leads to increased proliferation and decreased differentiation of CPC (50). PLK2 may also play a key role in dynamic compression enhanced chondrogenesis (51). In fibrotic diseases, the loss of PLK2 function leads to the transformation of fibroblasts into myofibroblasts, thus promoting the occurrence and development of the disease, the specific mechanism of which will be discussed later (22, 23, 52, 53).

### Neural Development

A large number of studies have focused on the role of PLK2 in the development and function of the nervous system. In the fourteenth day of rat embryonic development, PLK2 expresses in cortical plate, rather than the ventricular/subventricular zones (VZ/SVZ); In immature cortical neurons, PLK2 locates in the cell body and dendrites, and is upregulated by brain-derived neurotrophic factor (BDNF) and downstream ERK signaling pathway, which is necessary for BDNF to promote dendritic growth. Deletion of PLK2 affects dendrite development in a dose-dependent manner (54). PLK2 and poliovirus receptors (PVR) are essential for neuronal differentiation driven by nerve growth factor (NGF) and are negatively regulated by alphaB-crystallin (Cryab); Silencing PLK2 or PVR could block neuronal differentiation induced by NGF (55).

Homeostatic synaptic depression (HSD) is the homeostasis compensation mechanism for increased neural network activity, including loss of some excitatory synapses to reduce excitability and subsequent downscaling of the remaining synapses to further enhance homeostasis (56). Mounting studies have shown that excessive activation of hippocampal neurons induces the expression of PLK2, leading to the degradation of the spine associated RapGAP (SPAR), and feedback reduction of neuronal excitability (57–60). CDK5 activates phosphorylation of PLK2 binding sites in SPAR (a kind of Rap suppressor), then leads to PLK2 recruitment and accumulation (57). Activated PLK2 is highly phosphorylated, and its phosphorylation sites could regulate PLK2 kinase activity, in which S299 and S588 are involved. Mutations at sites above of PLK2 (S299E, S588A, and S588E) in neurons result in extreme activation of their anti-SPAR ability and impairment of the dendritic spines stability of primary hippocampal cells (61). A multi-subunit E3 ubiquitin ligase (Skp1/Cul1/F-box protein complex, SCF) is involved in ubiquitination degradation of SPAR, and blocking SCF might block PLK2-dependent SPAR degradation (62). In addition, over-activity induced PLK2 also directly eliminates Ras agonist RasGRF1 through phosphorylation mediated ubiquitination degradation, and PLK2 phosphorylation stimulates Ras inhibitor SynGAP and Raf agonist PDZ-GEF1. PLK2 comprehensively regulates these factors, contributing to maintain the homeostatic plasticity (60).

PLK2 directly binds to n-ethylmaleimide-sensitive fusion protein (NSF) in an ATP-dependent manner, disrupting its interaction with AMPA receptor GluA2 subunit, promoting extensive loss of GluA2 on the surface of rat hippocampal neurons and reducing AMPAR current and surface stability of synapses (59). SynGAP, a postsynaptic GTPase activating protein (GAP), is abundant in the postsynaptic density (PSD) scaffold, of which PSD-95 is the most prominent. Phosphorylation of synGAP-α1 by PLK2 and Ca2^+^/calmodulin-dependent protein kinase II (CaMKII) significantly reduces its binding to PDZ domain in PSD-95. These PDZ domains are occupied by other proteins, which changes the composition of PSD. This change may be as important as the reduction of synaptic Ras/Rap GAP activity in the pathological process of autism or epilepsy (63). PLK2 co-regulates synGAP kinase activity with CDK5 and CaMKII. After Ca2^+^/CaM is added to synGAP’s PLK2 phosphorylation system, the combination of Ca2^+^/CaM with synGAP causes conformational changes, increasing the availability of CDK5 and PLK2, accelerating kinase reaction, and phosphorylating additional residues. PLK2 phosphorylated synGAP is more likely to inactivate Ras, resulting in a relative increase in Rap and promoting the endocytosis of synaptic membrane AMPAR. PLK2 and CDK5 work together to activate the Rap pathway by triggering SPAR removal and increase GAP activity of r-synGAP on HRas, driving synaptic AMPAR elimination (64). In enhanced hippocampal activity induced by GABA receptor antagonists, upregulated PLK2 also acts as a downstream molecule of miR-134-Pum2 to maintain synaptic homeostasis (56). On the other hand, PLK2 interacts strongly and directly with the actively-induced amyloid precursor protein (APP), promoting APP phosphorylation (T668/S675) and amyloidopathy. It affects neurohomeostasis and is involved in the pathological process of Alzheimer’s disease (AD) (65). Fear Condition has further confirmed that PLK2 plays an important role in maintaining synaptic plasticity (66).

Ras promotes long-term potentiation (LTP), whereas Rap mediates long-term depression (LTD) (67, 68). PLK2 regulates Ras and Rap by regulating RasGRF1/SynGAP and SPAR/PDZ-GEF1 and has significant effect on memory formation (60). Interference with PLK2 function disrupts the homeostasis adaptation of synapses to enhanced activity and impaired behavior adaptation during various learning tasks (69). The activity dependent transcription factor Npas4 aims directly on the promoter and enhancer regions of PLK2, and conditional knockout of Npas4 in hippocampal neurons results in a significant decrease in PLK2 expression, preventing the formation of context memory and the learning-induced synaptic modification. Overexpression of PLK2 can restore memory formation and normal behavior in experimental animals (70). In a rodent model of hypoxia-induced neonatal seizures, after initial upregulation, AMPA receptor function of hippocampal CA1 pyramidal neurons shows transient attenuation, which is consistent with the transient increase in PLK2 expression and function. One week later, the function of AMPA receptor is up-regulated again, while the expression and function of PLK2 are negatively regulated by increased mTOR (71). Prenatal stress decreases the density of dendritic spines and impairs the LTP in the hippocampus of young rats. The number of NR2B and NR2A subunits decreases, while the postsynaptic scaffold proteins PSD-95 and SPAR also decrease, and PLK2 and SCF ubiquitin ligases increase, promoting ubiquitination and degradation of SPAR (72).

## Role of PLK2 in Basic Pathophysiological Processes

### Oxidative Stress and Endoplasmic Reticulum Stress

PLK2 is an important molecule in response to various stresses. PLK2 induced by oxidative stress in cells with abnormal mitochondrial function, mediates glycogen synthase kinase (GSK) 3β phosphorylation and promotes NRF2 nuclear translocation, preventing p53 induced cell death and promoting cell survival (73). In oxidative stress-induced glaucoma, up-regulated PLK2 provides protection to retinal ganglion cells also through this mechanism (74). Loss of synthesis of cytochrome c oxidase 2 (SCO2) impairs mitochondrial respiration, while expression of PLK2 elevates to make cell survive (75). In the treatment of protocatechuic aldehyde (PCA) to Parkinson’s disease (PD) induced by 1-methyl-4-phenyl-1,2,3,6-tetrahydropyridine (MPTP), PLK2 inhibition or knockdown eliminates the protection of PCA to improve mitochondrial membrane potential (MMP), mitochondrial complex I activity and reactive oxygen species (ROS) level, while overexpression of PLK2 enhances the protection of PCA in PD model (76). However, the alternative view is that PLK2 is involved in ROS-induced cell death. Celastrol induced ROS promotes p53 phosphorylation and p53-dependent PLK2 expression and inhibits tumor survival (77). In diabetic nephropathy patients, PLK2 is upregulated, which mediates G1 phase arrest and induces apoptosis of podocyte cultured with high D-glucose (HDG). Both PLK2 knockdown and antioxidant N-acetylcysteine (NAC) inhibit ROS production and MMP reduction and promote cell survival. Cytotoxic effects of PLK2-mediated HDG are associated with increased p53 expression and caspase-3 activation, relying on inflammatory cytokines such as TNF-α, IL-6, IL-1β, COX-2 and CXCL1 (78). At the same time, PLK2 expression is upregulated during cell stress induced by ischemia-reperfusion injury, leading to cell death through nuclear factor (NF) -κB signaling (21, 79).

Effect of PLK2 on endoplasmic reticulum (ER) stress is also controversial. It is reported that ER stress could induce PLK2 expression and lead to cell death (80). But more studies have suggested that PLK2 inhibits apoptosis and promotes survival by interacting with ER stress signals. For example, interference with PLK2 might lead to the loss of interaction with miR-101-3p target gene SKP1, and the accumulation of cotransfected overexpressed α-Syn protein due to decreased ubiquitination degradation, leading to ER stress of neurons, suggesting that PLK2 could prevent ER stress (28). PLK2 is hyper-expressed in multiple myeloma (MM) patients; PLK2 further inhibits C/EBP homologous protein (CHOP) and enhances inositol-requiring enzyme 1α (IRE1α) by inhibiting KIRA8 (kinase-inhibiting RNase attenuator 8), which in turn affects ER stress and facilitates cell survival; Meanwhile, KIRA8/IRE1α could reversely regulate PLK2 expression; KIRA8 and PLK2 inhibitors exert anti-MM effects by inducing apoptosis and regulating cell proliferation (81). In ER stress induced by Brefeldin A (BFA), increased binding of CHOP to the PLK2 promoter C/EBPα response element results in downregulation of PLK2 expression; Overexpression of exogenous PLK2 could inhibit cell apoptosis and promote cell proliferation (82). However, according to the limited results available, PLK2 plays an important role in coping with stress induced by exogenous cellular stimuli; Survival or apoptosis should depend on the intensity of exogenous stimulation and the degree of cell damage. When mild stimulation induces mild damage, PLK2 participates in the correction of adverse effects caused by stress; On the contrary, when severe stimulation induces severe damage difficult to correct, PLK2 directly leads to cell apoptosis/death.

### Viral Infection and Immune

PLK2 is upregulated in phytohemagluttinin (PHA) activated canine T cells, indicating PLK2 takes part in immune cell activation (83). In lipopolysaccharide (LPS) induced inflammation, expression of PLK2 is elevated, phosphorating a disintegrin and metalloprotease 17 (ADAM 17) and leading to release of tumor necrosis factor (TNF) receptor and pro-TNFα on cell membrane; Inhibition of PLK2 results in reduction of LPS-induced ADAM17-mediated pro-TNFα release from primary macrophages and dendritic cells (DCs) (84). In antiviral innate immunity, retinoic acid-inducible gene I (RIG-I)-like receptors (RLRs), RIG-I, and melanoma differentiation-associated gene 5 (MDA5) regulate transcription of type I interferon (IFN) and inflammatory cytokines by activating IFN regulatory factor (IRF) 3 and NF-κB; Knockout of the RNA-binding protein HuR, which could enhance IFN-β promoter activity and bind to the 3’ untranslated region of PLK2 mRNA to increase its stability, results in a significant decrease in PLK2 expression and IFNB1 expression after RLR stimulation. PLK2 deficient cells also shows reduced IRF3 nuclear translocation and IFNB mRNA expression during RLR signal transduction. These results suggest that HuR might promote RLR mediated IRF3 nuclear translocation and subsequent antiviral innate immune mechanism by maintaining PLK2 mRNA stability (85). Pan-PLK inhibitor BI 2536 treatment results in significant inhibition of antiviral genes (e.g., Cxcl 10 and IFNB1) expression and IRF3 nuclear translocation (86). Functional redundancy exists between PLK2 and its family member PLK4 (8, 40, 87). Therefore, antiviral gene expression decreases dramatically after simultaneous knockout of PLK2 and PLK4; And PLK2 is essential for viral sensing of DCs (86). Thus, it appears that PLK2 plays an active role in host antiviral immunity. Nevertheless, PLK2 could work adversely by promoting viral integration and replication. For example, in the infection of foamy virus (FV) with retroviruses and hepadnaviruses in its replication strategy, PLK2 interacts with prototype FV (PFV) to promote efficient integration of the PFV genome into the host chromatin, ensuring successful viral replication and transmission in cell cultures (88). Besides, avian metapneumovirus subtype C (aMPV/C) infection leads to upregulation of PLK2 in mammalian cells. Inhibition of PLK2 could reduce ROS production and p53-dependant apoptosis induced by aMPV/C, and decrease the virus release, suggesting that the high expression of PLK2 is associated with aMPV/C-induced apoptosis and viral replication (89). Contrasting to its family members, PLK2 is poorly studied in viral infection, and the exact role and mechanism remain unclear.

## Role of PLK2 in Diseases

### Hematological Neoplasma

Like its compatriots, the role of PLK2 in tumor has attracted considerable attention. Although it is reported that PLK2 is highly expressed in MM and facilitates tumor cell vitality by inhibiting KIRA8 induced CHOP mediated apoptosis (81), more studies have showed PLK2 acts as a tumor suppressor in hematological neoplasma. In B-cell lymphoma (26), acute myelogenous leukemia (AML) and myelodysplastic syndromes (MDS) (90), remarkable reduction of PLK2 expression might be related to abnormal methylation. As in B-cell lymphoma, abnormal methylation occurs in the CpG island of the PLK2 gene; The PLK2 expression of DG75 (EBV-) and Rael (EBV+) cell lines increase after demethylation with 5-AZA and is further upregulated by combined administration of histone deacetylase inhibitor TSA. Methylation and expression silencing occur in both p53 wild-type and mutant cell lines, suggesting that the methylation of PLK2 in B cell derived tumors is independent of p53. In contrast, B cell mitogens is able to induce PLK2 expression and re-expression of PLK2 could lead to apoptosis (26). While in AML and MDS, PLK2 is similarly methylated, although the PLK2 methylation status has no significant effect on clinical indicators and long-term prognosis (90); Additionally, in myeloproliferative neoplasm (MPN) like MDS, the disordered co-expression and disrupted signal transduction of PLK2 with myeloid tumor suppressor Egr1 and JunB may be a pathogenesis (91). Even in recent MM studies, PLK2 was identified a methylation gene independent of CpG island (92). It also suggests that there is a complex relationship between various pathogenic mechanisms. As an example, in B-cell tumor, there is functional redundancy between PLK2 and PLK3, and the decline of PLK2 expression is always accompanied by the overexpression of PLK3 (93).

In B cell chronic lymphocytic leukemia (B-CLL), the expression of PLK2 is correlated with the efficacy of purine nucleoside therapy. PLK2 hyper-expressed patients shows higher cytotoxicity, revealing that PLK2 might inhibit B-CLL (94). MiR-126 is involved in inflammation, angiopoiesis, and thus tumorigenesis (95). The cross-talk between miR-126 and PLK2 in hematological neoplasma is also receiving increasing attention. MiR-126 could inhibit apoptosis of AML cells and enhance cell viability and PLK2 exerts anti-tumor effects through negatively regulating of miR-126 (96). PLK2 expression is downregulated in AML, while expression of p-ERK, p-MYC and total MYC, which are critical for the survival of inv(16) leukemia-initiating cells and AML cells, is increased. These effects are reversed after miR-126 knockdown (97).

### Solid Organ Tumor

PLK2 is reported to be a tumor suppressor in solid organ tumor as well. Its repressed expression is associated with overall survival in non-small cell lung cancer (NSCLC) patients (98). The measured tumor diameter of human PLK2 deficient NSCLC cell xenograft is larger in mice; Interestingly, *in vitro* cell culture suggests that anti-tumor effect of PLK2 might result from a response to hypoxic tumor microenvironment (TME) (99). Compared to normal tissues and polyps, PLK2 expression is absent in colorectal cancer (CRC) (100). Also, in hepatocellular carcinoma (HCC), promotor methylation might be the reason of decreased PLK2 expression, and inhibition with siRNA could accelerate human HCC cell line growth (101). PLK2 is directly inhibited by significantly upregulated miR-27a in throat tumor, resulting in enhanced cell viability, promoted colony formation, and inhibited cell apoptosis (102). Circ_0102049 could heighten PLK2 expression by depressing miR-520g-3p, and inhibit proliferation, invasion, migration and cell cycle of osteosarcoma (OS) cell line MG63; PLK2 inhibiting leads to a significant elevation in tumor volume and weight in the MG63 cell xenograft mouse model (14). In glioblastoma multiforme (GBM), reduced PLK2 expression indicates treatment resistance and poor prognosis; overexpression of PLK2 could repress the tumor characteristics of GBM cell and lower the incidence of acquired TMZ resistance (103). And in epithelial ovarian cancer (EOC), CpG island methylation caused PLK2 downregulation was related to paclitaxel and platinum tolerance and postoperative recurrence, being confirmed by knockdown and overexpression experiments and indicating the relevance to G2-M arrest (104, 105). Besides, PLK2 collaborates with other tumor suppressor genes (TSG) (TGFBI, PTEN, LZTS1, ING4, CDKN1A, ING1, hEx, and FBW7 etc.) in the generation of paclitaxel resistance (106). p53 dependent PLK2 expression, resulting from celastrol induced ROS production, might increase the apoptosis of G1 subgroup and suppress breast cancer MCF-7 cell viability *via* pro-apoptotic poly(ADP-ribose) polymerase-2 (PARP-2) (77). The tumor-inhibiting effect of PLK2 might also be related to the mammalian target of rapamycin (mTOR) signaling pathway. p53 dependent PLK2 interacts with TSC1/2 to amplify their suppressive effect on mTOR; Loss of PLK2 function promotes CRC and NSCLC progression (99, 100); and MSI-H specific frameshift mutation may be the internal cause of PLK2 dysfunction (107).

For all this, the role of PLK2 in solid organ tumor is still elusive and more reports indicate PLK2 could exacerbate tumor progression. DNA damage and S-phase checkpoint defects in PLK2-deficient human tumor cells caused by replication stress eventually leads to increased cell death, suggesting that PLK2 plays an important role in maintaining stable replication and cell survival of human tumor cells (108). Different from EOC, PLK2 protein level elevates markedly as a result of low promotor methylation (25). Its expression is positively related to the malignancy of gliomas while high expression indicates a poor prognosis (27, 103, 109). In PLK2^-/-^ triple-negative breast cancer patient-derived xenograft (PDX) mice model, re-expression of PLK2 significantly reduces the therapeutic effect of PLK1 inhibitor Volasertib (110).

PLK2 promotes tumor through complex regulatory mechanisms. Hyper-expressed PLK2 in CRC binds to Fbxw7 and leads to its degradation, stabilizing Cyclin E and facilitating cell vitality (111); And this regulation targeting to Fbxw7/Cyclin E is negatively controlled by tazarotene-induced gene 1 (TIG1) (112). Higher expression of PLK2 in proximal CRC is associated with mismatch repair defects, B-raf serine/threonine kinase proto-oncogene and Kirsten rat sarcoma virus oncogene homologous mutations, suggesting more chemotherapy resistance and worse prognosis for patients receiving chemotherapy (113). Elevation of PLK2 is also positively related to FOXD1; PLK2 knockdown causes restrained proliferation and increases apoptosis in FOXD1 overexpressed HT29 cells (114). PLK2 is also regulated by Hedgehog (Hh) signal. Inhibition of Hh signal leads to reduction of PLK2, degradation of anti-apoptotic myeloid cell leukemia 1 and cell apoptosis in cholangiocarcinoma cells (115). Additionally, PLK2 is negatively correlative to Notch signal (103, 109), and could be ubiquitin-dependently degraded in the presence of E3 ubiquitin ligase RNF180 (27).

There are feedback regulations between PLK2 and p53. Mutation of TP53 in CRC lowers PLK2 expression (113). Meanwhile, PLK2 binds and phosphorates mutated p53, enhancing its carcinogenic activity; Regulation of PLK2 by wild-type or mutated p53 results in tumor cell growth inhibition or cell proliferation enhancement and chemotherapy resistance respectively; siRNA of mutated p53 or PLK2 improves treatment outcome (116). Phosphorylation of p53 family member TAp73 at Ser48 restricts its nuclear translocation and anti-tumor effects, which could be reactivated by dephosphorylation; Contrasting to cisplatin alone, combination therapy with PLK2 inhibitor (ELN582646) upregulates p21 and puma expression in head and neck squamous cell carcinoma and OS cell line (117, 118); Inhibiting PLK2 in TAp73-rich OS cell line Saos2 leads to reduced cell proliferation, increased apoptosis, and decreased invasion; However, these changes are not observed in TAp73 KO Saos2 (119, 120). Osteoblastic OS expresses higher TAp73 and PLK2 than chondroblastic OS, indicating poor differentiation and prognosis; Abundant TAp73 in Saos2 and OS PDX mice promotes PLK2 expression, affecting osteopontin (OPN) and osteocalcin (OCN) and calcium deposit; PLK2 silencing prevents PDX-OS cell colony formation, facilitates cisplatin sensitivity, and improves curative effects (121). Thus, p53 family is deeply involved in the tumor promotion of PLK2.

Reportedly, PLK2 expression was positively correlated to paclitaxel resistance resulting from its anti-proliferative effects during mitosis in ovarian cancer cell line A2780 and promoting tumor cell viability (122). Different from Syed et al. (104), the difference in chemotherapeutic drug resistance pattern may be responsible for the difference in the influence of PLK2. TP53 deletion or mutation has similar effects on promoting tumor cell apoptosis induced by paclitaxel and enhancing drug sensitivity as PLK2 silencing with siRNA (37). The contradiction between expression and function was also observed in gastric cancer; PLK2 was overexpressed in SGC-7901 cell line, while silencing of PLK2 could further promote the growth of SGC-7901 cell by inhibiting apoptosis (apoptosis-related genes Bax and caspase 3 were down-regulated at the protein level) (123). And, in SGC-7901, PLK2 might be inhibited by anti-tumor miR-126; But PLK2 was still identified tumor suppressive in SGC-7901, because the tumor inhibition of miR-126 might be a symphonic regulation of PLK2, PI3KR2 and Crk; The limitation of this study was the lack of direct intervention on these effectors in SGC-7901 cell line to clarify their exact role (124). However, as a potential therapeutic target, the role of PLK2 in tumors and its relationship with chemotherapy sensitivity need further exploration

### Parkinson’s Disease

In PD, the number, distribution and phosphorylation state of α-Synuclein (α-Syn) affect the progression of the disease. α-Syn is a soluble presynaptic protein that is low expressed under normal physiological conditions and is associated with dopamine uptake, synaptic plasticity, and vesicle maintenance (125). In and ex vivo study revealed that α-Syn significantly inhibited tyrosine hydroxylase (TH), and its overexpression could activate protein phosphatase 2A (PP2A) (126). α-Syn accumulation is related to inflammation and cell death, enhancing PLK2 and GSK3β activities, and increasing phosphorylated α-Syn and Tau levels (127). In estrogen-related receptor gamma (ERRγ) overexpressed SHSY5Y cells, PLK2 is upregulated, participating GSK3β phosphorylation, inducing synapse upscaling, and improving dopaminergic neuron characteristics (upregulation of tyrosine hydroxylase, dopamine transporter and vesicle monoamine transporter 2) (128). Endogenous GSK3β activity might affect PLK2-mediated regulation of α-Syn (129). The expression of Tau protein is also correlated with the significant increase of PLK2 level, which could activate different kinases, leading to the phosphorylation of Tau and other proteins (including α-Syn), and result in the development of PD (130). PLK2 is regulated by ubiquitination degradation (28, 127). Overexpression of the conserved E3 ubiquitin ligase Parkin (synergistic with E1 activase and E2 binding enzyme) activates the ubiquitination, reducing PLK2, PARP, caspase-3 and CD3δ levels, and promoting α-Syn degradation (131–133). α-Syn-PLk2-ROS signaling pathway is involved in PD with insulin resistance (134).

In cell line and primary culture, inhibiting PLK2 increases α-Syn in presence of GSK3β (129). Kinase activity of PLK2 could suppress α-Syn toxicity and eliminate it through autophage, protect TH+ neurons, and inhibit Neurodegeneration as well as hemiparkinsonian motor symptoms; The PLK2/α-Syn co-overexpression by Stereotaxic injection results in symmetrical rats’ forelimbs, while loss of PLK2 kinase activity leads to impaired opposite forelimb activities (135). Phosphorylation at Ser129 is not necessary for PLK2 reducing α-Syn but macroautophagy (136). PLK2 interacts with N-terminal of α-Syn, forming a protein complex degraded through macroautophagy; inhibition of autophagy leads to α-Syn accumulation and PLK2 elevation; PLK2 overexpression decreases α-Syn in HEK-293T and multi-ubiquitination also plays its role (137). So, it is considered PLK2 possesses dual kinase/chaperone activity (138). On the contrary, presynaptic total and phosphorylated α-Syn decreases after BI 2536 inhibition, while aggregation of α-Syn does not change; But both phosphorylation and aggregation decrease after PLK2 KO, preventing neuropathy (139). Under iron overload, α-Syn expression and phosphorylation (Ser129) are increased, and PLK2 and Casein kinase 2 (CK2) are upregulated (140). In MPTP induced PD models, the expression of PLK2 is significantly upregulated, accompanied by increased levels of total, phosphorylated and oligomized α-Syn, and decreased levels of PP2A, TH, and dopamine transporter (DAT) (reflecting the function/number of dopaminergic neurons) (141). Therefore, PLK2 exerts different effects on α-Syn under different research settings.

Although the regulation of total α-Syn by PLK2 is controversial, it could significantly alter the phosphorylation status of α-Syn and cause neurotoxicity (15, 16). Lewy bodies (LBs) resulted from α-Syn phosphorylation and polyaggregation is the major feature of PD, dementia with LBs and other neurological diseases (17). α-Syn overexpression is associated with reduced immune proteasome function, which in turn limits PLK2 degradation, exacerbates α-Syn phosphorylation and aggregation, and ultimately leads to neurodegeneration (142). PLK2 is a major kinase that catalyzes the phosphorylation of α-Syn at Ser129 in central nerve system (143, 144), and the conversion is efficient (>95% conversion) (145); But the membrane binding and internalization abilities of different α-Syn mutants and phosphorylated proteins are different (146). More than 80% of p-Ser129 α-Syn is co-located with PLK2. In addition, the number of double-positive cells in the substantia nigra cells of older monkeys is more than 3 times higher than that of adult monkeys, suggesting PLK2 might be closely related to the accumulation of p-Ser129 α-Syn induced by aging (147). Moreover, PD patients’ hippocampus with dementia contains more p-Ser129 α-Syn dramatically than without, revealing phosphorylated α-Syn exhibits strong neurotoxicity and plays a significant role in the development of PD (148). More phosphorylated and oligomerized α-Syn appears in sera or brain of PD patients and older monkeys, due to increased PLK2 and decreased PP2A expression. Phosphorylated α-Syn enters neuron, exacerbates PP2A activity decline, and promotes α-Syn phosphorylation and oligomerization (149, 150). Phosphorylation at Ser129 also regulates the inhibition of TH by α-Syn; PLK2 reduces its ability to inhibit TH or activate PP2A by phosphorylation of α-Syn (126). PLK2 mainly phosphorylates soluble α-Syn (151); Inhibition of PLK2 triggers autophagic elimination of α-Syn (152). Oxidative stress might play a key role in PLK2 phosphorylation of α-Syn, and antioxidant NAC could completely block iron-induced up-regulation of PLK2, CK2 and p-Ser129 α-Syn (140); However, as PLK2 induces elevation of α-Syn in copper-treated SHSY5Y neuroblastoma cells, both PP2A level and oxidative status remains unchanged (153). Glutamate-mediated excitotoxicity is often considered as the mechanism of cell death in PD (154); Group II metabotropic glutamate receptors (mGLU2/3) are highly expressed in the preterminal region of subthalamic synapses, and activation of them could inhibit glutamate release from the presynaptic membrane (155, 156). In MPTP-induced PD, both expression and function of PLK2 are inhibited by mGLU2/3 (157).

Nevertheless, PLK2-induced α-Syn phosphorylation is not the only mechanism of neurodegeneration (158, 159); Transfection of PLK2 into the substantia nigra induced p-Ser129 α-Syn elevation does not lead to dopaminergic cell death neither (160). In PD, PLK2 could affect the expression, phosphorylation and aggregation of α-Syn, leading to neurotoxicity, impaired function and even death of dopaminergic neurons, and ultimately PD is still a widely held view. The use of PLK2 inhibitors to treat neurodegenerative diseases such as PD has become a possible option and will be reviewed below.

### Fibrotic Diseases

The essence of fibrosis is that under the action of various pathogenic factors (smoking and dust in lung, drinking in liver, hepatitis virus infection, ischemia in heart, etc.), relying on distinct trigger mechanism and subsequent activated signal pathways (mainly transforming growth factor-β, platelet-derived growth factor, WNT, and Hh), fibrous connective tissue is excessively deposited in target organs, causing organ remodeling, malfunction, or even failure (161). In this decade, the role of PLK2 in fibrotic diseases has attracted growing attention. A recent study suggested that PLK2 KO fibroblasts exhibited higher spontaneous myofibroblast differentiation, reduced proliferation rate, and overexpression of pro-fibrotic OPN (53). PLK2 expression decreases in patients with pulmonary fibrosis; Primary fibroblasts with PLK2 KO shows myofibroblast phenotype; The expressions of OPN, IL-18, ACTA2, COL1A1 and COL3A1 in the lung tissues of PLK2 KO mice are significantly increased; And drug inhibition of PLK2 in human lung fibroblasts leads to a fibrotic phenotype (52). PLK2 is upregulated as a node gene 7 days after acute myocardial infarction, and interacts with Rasl11b, Atxn10, Myl12B-Rock2 etc. to participate myocardial remodeling (162). Promoter methylation induced by hypoxia results in a 50% decrease in PLK2 expression in atrial fibrillation (AF) patients; In canine tachycardia, PLK2 expression is decreased in tissues of atria, but not ventricles; Drug inhibition or KO of PLK2 leads to cardiac fibroblasts displaying myofibroblast phenotype; PLK2 KO mouse heart fibroblasts secretes inflammatory OPN; The concentration of OPN in peripheral blood of AF patients with myocardial fibrosis is significantly higher than that of patients with sinus rhythm and AF patients without fibrosis. PLK2 KO mice might serve as a model of diastolic heart failure, showing left ventricular diastolic dysfunction, tachycardia, and typical fibrotic surface electrocardiogram abnormalities (PQ and QRS prolonged) (22, 23). In terms of mechanism, ERK1/2 signaling pathway is the molecular association between the decrease of PLK2 expression and the upregulation of OPN (22, 23, 53).

## Prospect of PLK2 Application in Disease Diagnosis and Treatment

Since PLK2 is extensively and deeply involved in the basic life activities of cells and the occurrence and development of diseases, its expression level may have predictive significance in the diagnosis and prognosis of diseases. The expression of PLK2 increases in people with high formaldehyde exposure, which can be used as an indicator of formaldehyde exposure (163). PLK2 is overexpressed in bladder cancer, and quantitative analysis of urine has showed that it is also associated with transitional cell carcinoma, which is predictive with a sensitivity of 80% and a specificity of 64% (164). Additionally, a low level of PLK2 expression indicates a poor prognosis for patients treated with radiation therapy after breast-conserving surgery (165).

In the therapeutic field, 5-ASA may be a valuable new drug target for the prevention and treatment of AF fibrosis and diastolic heart failure by restoring physiological PLK2 expression and blocking OPN release (22, 23). Cell internalization could be realized by the preparation of nanoparticle encapsulated PLK2 using the total recirculating one machine system (TROMS). In addition, the phosphorylation activity of PLK2 at α-Syn Ser129 is maintained. And a drug delivery system (DDS) has been constructed for continuous delivery of PLK2 into cells, which is conducive to further study of the biological effects of PLK2 on dopaminergic neurons (166).

Inhibition of PLK2 is also a potential treatment option for many diseases. PLK2 inhibitors at therapeutic doses are not genotoxic and are safe and effective (118). The PLK2 specific inhibitors C2 and C21 constructed based on tetrahydropteridin effectively inhibit the growth of various human tumor cell lines *in vitro* (167). PLK2 specific inhibitor 7AO (ON1231320) blocks tumor cell cycle during mitosis, leading to cell apoptosis; Synergistic action with paclitaxel effectively suppressed tumor growth *in vivo* (168). In neurodegenerative diseases, oral administration of potent selective inhibitors of PLK2 that could cross the blood-brain barrier significantly reduces the phosphorylation of α-Syn in rat brain, providing a direction for the treatment of PD (117). Isorhamnetin-3-O-β-D-glucoside (IR3G) could bind and inhibit PLK2 with high affinity and may inhibit macrophage function and exert strong anti-inflammatory activity, as well as combat neurotoxicity and motor loss induced by 6-OHDA in SHSY5Y cells (169). Oral administration of PLK2 inhibitor based on dihydropteridinone reduces p-Ser129 α-Syn in the cerebral cortex of rats by about 41-45% (170). PLK2 also plays a pathologic role in the pathogenesis of AD, promoting the production of Aβ *in vivo*; Drug inhibition of PLK2 prevents the formation of Aβ, synaptic loss and memory decline in AD mouse models (171). In addition, calcipotriol inhibits the proliferation of keratin forming cells by inhibiting PLK2 in the treatment of psoriasis (172). Inhibition of PLK2 promotes synovial cell apoptosis, alleviates synovial injury, and prevents cartilage injury and chondrocyte apoptosis to treat knee osteoarthritis (18).

## Conclusion

As mentioned above, PLK2, a member of the evolutionarily conserved PLK family, is extensively and deeply involved in the normal physiological activities, the stress response to external stimuli and the development and progression of diseases. In some aspects, the role of PLK2 is relatively clear. For example, normal PLK2 expression and function are necessary for the normal operation of cell cycle, and PLK2 is also involved in regulating cell differentiation and maintaining the stability of nervous system function. In addition, in hematological neoplasma, most of the current studies believe that PLK2 acts as a tumor suppressor, and the kinase activity of PLK2 is also involved in the pathological mechanism of neurodegenerative diseases such as PD. Meanwhile, there seems to be a negative relationship between PLK2 and the development of fibrotic diseases. However, the role and regulatory mechanism of PLK2 in the stress response to external stimuli and solid organ tumors development and progression are far from consensus. The essence behind many seemingly contradictory phenomena is the complexity of its function and interaction regulatory network and may also be due to the differences in experimental models and designs adopted by different studies. Nevertheless, some preliminary attempts to use PLK2 as a predictor and therapeutic target for disease have brought encouraging results and showed promising prospects. There will definitely be more studies focusing on PLK2, which will help us better understand PLK2 and make better use from principle to practice.

## Author Contributions

HL, CZ, and CN designed the research. CZ and CN drafted the manuscript. HL improved the structure of this manuscript. HL, CZ, and CN discussed and revised the manuscript. All authors contributed to the article and approved the submitted version.

## Conflict of Interest

The authors declare that the research was conducted in the absence of any commercial or financial relationships that could be construed as a potential conflict of interest.

## Publisher’s Note

All claims expressed in this article are solely those of the authors and do not necessarily represent those of their affiliated organizations, or those of the publisher, the editors and the reviewers. Any product that may be evaluated in this article, or claim that may be made by its manufacturer, is not guaranteed or endorsed by the publisher.
